# Supplementary material for: Explosive fragmentation of Prince Rupert’s drops leads to well-defined fragment sizes
Source: Nat Commun. 2021 May 4;12:2521. doi: 10.1038/s41467-021-22595-1 (PMC8097073; doi:10.1038/s41467-021-22595-1)
Supplement: Supplementary file 1 — Description of Additional Supplementary Files [file 41467_2021_22595_MOESM1_ESM.pdf]

Title: Supplementary Movie 1

Description: Fragmentation of a Prince Rupert’s drop, recorded at a frame rate of 10233 fps. The Prince Rupert’s drop has a width of approximately 5 mm.
