# Supplementary material for: Explosive fragmentation of Prince Rupert’s drops leads to well-defined fragment sizes
Source: Nat Commun. 2021 May 4;12:2521. doi: 10.1038/s41467-021-22595-1 (PMC8097073; doi:10.1038/s41467-021-22595-1)
Supplement: Supplementary file 2 — Supplementary information [file 41467_2021_22595_MOESM2_ESM.pdf]

Supplementary Information for: Explosive fragmentation of Prince Rupert's drops leads to well-defined fragment size

Stefan Kooij,[1,*] Gerard van Dalen,[2] Jean-François Molinari,[3] and Daniel Bonn[1]

[1] *Van der Waals-Zeeman Institute, University of Amsterdam, Science Park 904, Amsterdam, Netherlands*

[2] *Unilever Research and Development Vlaardingen, Olivier van Noortlaan, P.O. Box 114, 3130 AC Vlaardingen, The Netherlands*

[3] *Department of Materials Science and Engineering, École Polytechnique Fédérale de Lausanne (EPFL), 1015 Lausanne, Switzerland*

* email: s.a.kooij@uva.nl

Supplementary Note 1: Origin of fragments inside a Prince Rupert's drop. To assess the origin of the different fragments, we initiate the fragmentation of a Prince Rupert's drop encased inside a gel. The gel prevents the fragments from exploding outwards, making it possible to determine the structure of the fragmented drop. Because fragmentation is driven by internal stresses, we believe that there will be no significant effect of the gel on the fragmentation process. Supplementary Fig. 1 shows colour-labeled fragment sizes of a part of the broken Prince Rupert's drop. Fragments that belong to the left-hand side of the double exponential distribution, i.e. fragments of size < 300 μm (Fig. 1 (e)), seem to be spread evenly through the droplet. This indicates that these smaller fragments, also referred to as the remaining 'dust', originate from a secondary fragmentation process. The arrangement of the fragments does have a certain structure, which resembles the shape of a pine cone (Supplementary Fig. 2 (a)). This structure is due to the fact that the fractures follow the direction of the stress.

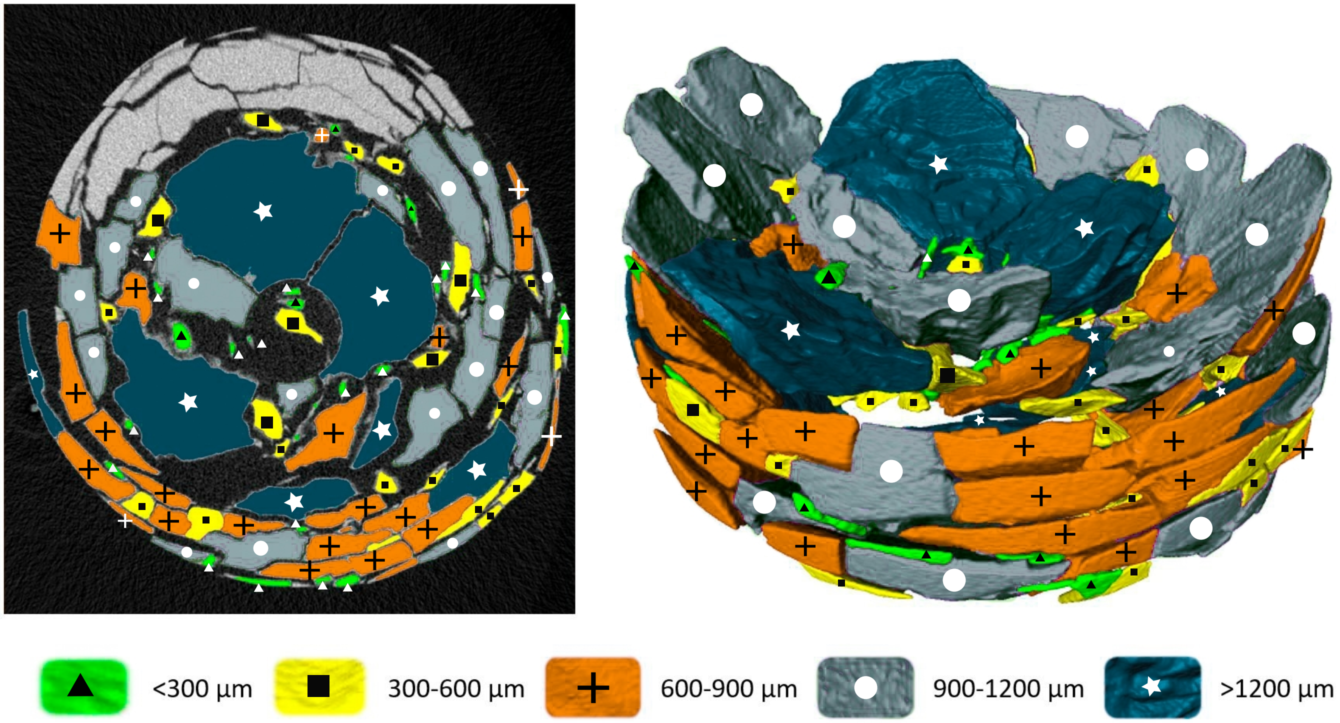

Supplementary Figure 1. **Origin of Prince Rupert's drop fragment sizes**. Colour-labelled fragments of a Prince Rupert's drop that has been broken inside a Carbopol matrix as determined by micro-computed tomography (micro-CT). Colours indicate size ranges: < 300 μm (Green), $300 - 600$ μm (Yellow), $600 - 900$ μm (Orange), $900 - 1200$ μm (Grey) and > 1200 μm (Blue). The Carbopol matrix keeps the fragments in place during fragmentation, making it possible to determine the origin of certain fragment sizes. The smallest fragments (< 300 μm), that belong to a separate exponential regime (Fig. 1 (e)), seem to have no specific origin, indicating that these fragments are due to some secondary fragmentation process.

Supplementary Note 2: Fracture speed. In the fragmentation of tempered glass plates, cracks accelerate until they become unstable and branch. After the crack branches, the crack speed has slowed down and starts to accelerates until it splits again. Supplementary Fig. 2 (a) shows an image sequence of the fragmentation of a tempered glass plate filmed with a high-speed camera, with 20 µs between frames. By looking at the distance travelled by the crack front we find that the average crack speed is around a third of the sound velocity in the glass (∼1500 $\mathrm{m\,s^{-1}}$), in accordance with what has been found in other studies[1,2]. By looking at more detailed high speed camera images (Supplementary Fig. 2 (b)) at a higher frame rate (3.8 µs between frames), we observe a clear acceleration of a crack between points x_1, x_2 and x_3. Between x_1 and x_2 the average velocity is about ∼1100 $\mathrm{m\,s^{-1}}$ and between x_2 and x_3 the average velocity is ∼2000 $\mathrm{m\,s^{-1}}$, almost twice as much. An even higher frame rate would allow measuring the whole acceleration profile between branch points; however this is beyond our technical capabilities.

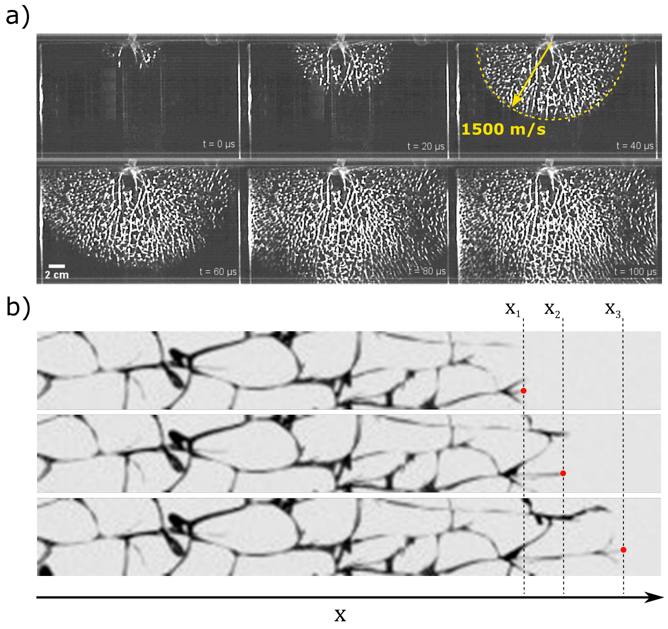

Supplementary Figure 2. **Crack propagation**. a) Image sequence of the fragmentation of a tempered glass plate with 20 µs between frames. The speed of the crack front, i.e. the average crack speed is about 1500 $\mathrm{m\,s^{-1}}$, roughly a third of the sound velocity in the glass, the same value as was found in a previous study[3]. b) Zoomed image sequence of the crack propagation of a tempered glass plate with 3.8 µs between frames. A clear acceleration of the crack after branching can be seen where the distance $|x_2 - x_1|$ is almost twice as large as $|x_3 - x_2|$. This demonstrates the typical acceleration of the cracks before they become unstable and branch.

Supplementary Note 3: Finite-element simulations. In[4] finite-element simulations of dynamic fragmentation of tempered glass were conducted. Tempered glass panels are subjected to high eigenstresses that induce a state of

compression along the surfaces and a state of tension in the inner part. Whenever a crack reaches the tensile region, it rapidly propagates and branches in all directions driven by the eigenstress (see Fig. 3 (b) in the main text). These mechanisms induce dynamic fragmentation and a distribution of fragment sizes (see for instance Fig. 15 in[4]).

Here, we perform the numerical results that were obtained through massively parallel simulations using the high-performance open-source software Akantu[5]. The simulations were launched on 192 cores on the EPFL Intel Xeon based computer cluster Deneb. The parallelism is based on a domain decomposition approach, in which the mesh is split into partitions, and each partition is transferred to a single processor. The synchronization is achieved with point-to-point communications along the partitions' borders of neighboring partitions.

The three-dimensional quadratic finite-element tetrahedral meshes with average element size of 0.3 mm, contained up to 20 Million degrees of freedom, with in-plane dimension 60 mm $\times$ 60 mm or 80 mm $\times$ 80 mm. The tempered glass plates thickness was varied between 3 mm to 8 mm. A parabolic profile of residual stresses was assumed throughout the thickness with a central tension varying depending on test cases between 35 MPa and 60 MPa[6–8]. Upon introduction of a 5 mm long notch on a side, explicit dynamics simulations reproduced the rapid crack growth and branching from this initial notch. The crack growth dynamics were captured using dynamically inserted cohesive elements. In this approach, the cohesive tractions gradually diminish in proportion to the crack opening according to a cohesive law. We have set the critical opening stress, upon which cohesive damage initiates, to a value of 70 MPa. A review of the basic principles of fracture mechanics and the cohesive approach to fracture can be found in[9]. The cohesive law parameters driving the crack dynamics were chosen to represent a brittle glass material and neglected rate dependency. The fracture energy was taken equal to 7.6 $\mathrm{J\,m^{-2}}$.

Naturally, the distribution of fragment sizes depends on plate dimensions and central tension and varies from case to case. For all cases, we have computed the average fragment size and normalized the corresponding fragment size distribution by this average fragment size. The Supplementary Fig. 3 (a) shows the ensemble average of all normalized fragment size distribution and agrees well with the experimental fragment sizes.

Supplementary Note 4: A comparison of Prince Rupert's drops data. Comparing our fragment size data of the Prince Rupert's drop to the earlier ones by Silverman et al.[10], we observe their data is substantially more noisy, but the results are in fact comparable (Supplementary Fig. 3). The noise in the data of Silverman et al. makes that they cannot clearly discriminate between a power law and an exponential behavior of the distribution. Moreover, as in the experiments of Silverman et al. several (50) drops of different sizes are mixed and measured, the sharp transition we observe between the two exponential regimes for a single is obscured, as this transition will depend on the initial

drop size.

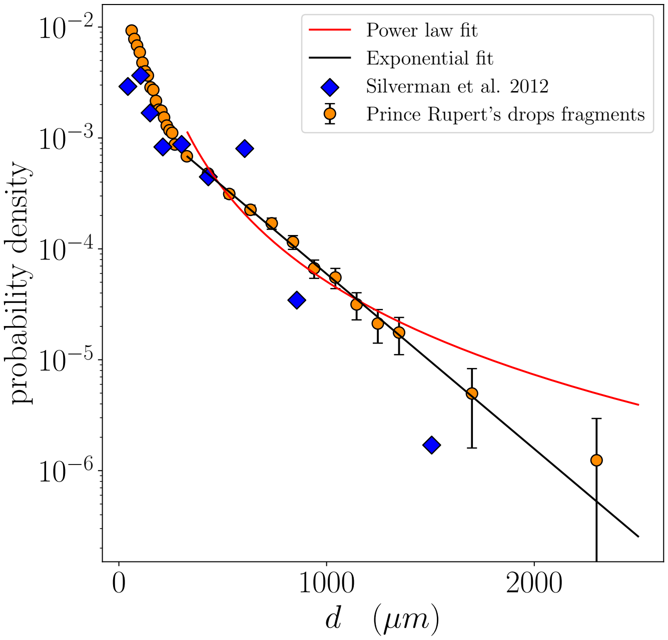

Supplementary Figure 3. **Power law versus exponential fitting**. Data of Silverman et al. compared with our fragment size data of the Prince Rupert drop[10], error bars indicate one SD. The data is comparable, though the data of Silverman et al. is too noisy for a clear discrimination between a power law and an exponential. Both an exponential and power law are fitted to the right hand side of the crossover, using a least square procedure. The fit parameters are $d = 0.28$ mm for the exponential and $k = 2.4$ for the exponent of the power law. Note the fit value of the exponential is slightly different from the fit in Fig. 1 (d), which is $d = 0.31$ mm, as this fit does not include the second exponential regime. Though it is quite clear from the graph that this part is better fitted with an exponential function than a power law, this can also be seen by applying the Akaike information criterion (AIC). To do so we calculate the maximum likelihood estimators (MLE), which yield $d = 0.28$ mm for the exponential and $k = 2.7$ for the power law. The maximum likelihoods then give that the exponential is far more probable to minimize the information loss and is therefore is the preferred model. Besides providing a better fit, the fit parameter in the exponential case can be derived from theory, which is not the case for the power law. Source data are provided as a Source Data file.

I. SUPPLEMENTARY REFERENCES

[1] G. Molnár, M. Ferentzi, Z. Weltsch, G. Szebényi, L. Borbás, and I. Bojtár, Fragmentation of wedge loaded tempered structural glass, Glass Structures & Engineering **1**, 385 (2016).

[2] P. Acloque, La fracture du verre propagation: influence des précontraintes, Verres Refract **17**, 151 (1963).

[3] J. H. Nielsen, J. F. Olesen, and H. Stang, The fracture process of tempered soda-lime-silica glass, Experimental mechanics **49**, 855 (2009).

[4] M. Vocialta, M. Corrado, and J.-F. Molinari, Numerical analysis of fragmentation in tempered glass with parallel dynamic insertion of cohesive elements, Engineering Fracture Mechanics **188**, 448 (2018).

[5] Akantu's user manual, `http://lsms.epfl.ch/akantu`, 2014.

[6] E. Mognato, A. Barbieri, M. Schiavonato, M. Pace, and S.-S. S. del Vetro-Venezia, Thermally toughened safety glass: correlation between flexural strength, fragmentation and surface compressive stress, Glass Performance Days , 115 (2011).

[7] K. Akeyoshi and E. Kanai, Mechanical properties of tempered glass, in *Proceedings of the 7th International Glass Congress*, Vol. 14 (1965) pp. 80–85.

[8] P. D. Warren, Fragmentation of thermally strengthened glass, Ceramic Transactions(USA) **122**, 389 (2000).

[9] T. L. Anderson and T. L. Anderson, *Fracture mechanics: fundamentals and applications* (CRC press, 2005).

[10] M. Silverman, W. Strange, J. Bower, and L. Ikejimba, Fragmentation of explosively metastable glass, Physica Scripta **85**, 065403 (2012).
